# Metabolic engineering of the cellulolytic thermophilic fungus *Myceliophthora thermophila* to produce ethanol from cellobiose

**DOI:** 10.1186/s13068-020-1661-y

**Published:** 2020-02-01

**Authors:** Jinyang Li, Yongli Zhang, Jingen Li, Tao Sun, Chaoguang Tian

**Affiliations:** 1 Key Laboratory of Systems Microbial Biotechnology, Tianjin Institute of Industrial Biotechnology, Chinese Academy of Sciences, Tianjin, 300308 China; 2 University of Chinese Academy of Sciences, Beijing, 100049 China

**Keywords:** *Myceliophthora thermophila*, Ethanol, Cellobiose, RNA-seq, Sugar uptake, Metabolic engineering

## Abstract

**Background:**

Cellulosic biomass is a promising resource for bioethanol production. However, various sugars in plant biomass hydrolysates including cellodextrins, cellobiose, glucose, xylose, and arabinose, are poorly fermented by microbes. The commonly used ethanol-producing microbe *Saccharomyces cerevisiae* can usually only utilize glucose, although metabolically engineered strains that utilize xylose have been developed. Direct fermentation of cellobiose could avoid glucose repression during biomass fermentation, but applications of an engineered cellobiose-utilizing *S. cerevisiae* are still limited because of its long lag phase. Bioethanol production from biomass-derived sugars by a cellulolytic filamentous fungus would have many advantages for the biorefinery industry.

**Results:**

We selected *Myceliophthora thermophila*, a cellulolytic thermophilic filamentous fungus for metabolic engineering to produce ethanol from glucose and cellobiose. Ethanol production was increased by 57% from glucose but not cellobiose after introduction of *ScADH1* into the wild-type (WT) strain. Further overexpression of a glucose transporter GLT-1 or the cellodextrin transport system (CDT-1/CDT-2) from *N. crassa* increased ethanol production by 131% from glucose or by 200% from cellobiose, respectively. Transcriptomic analysis of the engineered cellobiose-utilizing strain and WT when grown on cellobiose showed that genes involved in oxidation–reduction reactions and the stress response were downregulated, whereas those involved in protein biosynthesis were upregulated in this effective ethanol production strain. Turning down the expression of *pyc* gene results the final engineered strain with the ethanol production was further increased by 23%, reaching up to 11.3 g/L on cellobiose.

**Conclusions:**

This is the first attempt to engineer the cellulolytic fungus *M. thermophila* to produce bioethanol from biomass-derived sugars such as glucose and cellobiose. The ethanol production can be improved about 4 times up to 11 grams per liter on cellobiose after a couple of genetic engineering. These results show that *M. thermophila* is a promising platform for bioethanol production from cellulosic materials in the future.

## Background

Lignocellulose, which is mainly composed of polymers of glucose and xylose, is the most abundant carbohydrate resource on earth and is a major renewable feedstock for biorefinery [1]. Conversion of lignocellulose into biofuels such as ethanol is an important strategy for sustainable development. Extensive efforts have been made to engineer microorganisms to produce biofuels or chemicals from lignocellulose. However, the high costs of cellulose-to-sugar conversion and inefficient fermentation of both hexose and pentose sugars have made commercial production economically unattractive. Plant biomass hydrolysates consist of approximately 70% cellodextrins and glucose and 30% xylose [2]. Hydrolysis of cellulose by fungal cellulases primarily generates cellobiose, which can be further hydrolyzed to glucose by *β*-glucosidases. Therefore, organisms capable of efficiently utilizing cellodextrins and glucose are desired to produce biofuels from cellulosic biomass [3]. However, a high concentration of glucose inhibits cellulase activity and the fermentation of non-glucose sugars present in cellulosic hydrolysates because of carbon catabolic repression (CCR). Therefore, direct fermentation of cellodextrins is a better approach. The intracellular hydrolysis of cellobiose can relieve the effect of CCR on the utilization of other sugars during co-fermentation. However, *S. cerevisiae* cannot ferment the cellodextrins that are naturally released by cellulases. To engineer strains capable of fermenting cellobiose, researchers have introduced various heterologous pathways into *S. cerevisiae.* Such pathways include cellobiose utilization pathways consisting of a cellobiose transporter system (CDT-1 or CDT-2 from *N. crassa*) and either a hydrolytic enzyme (GH1-1, *β*-glucosidase from *N. crassa*) or a phosphorolytic enzyme (CBP, cellobiose phosphorylase from *Saccharophagus degradans*) [4–6]. The initially engineered *S. cerevisiae* strain had a minimal synthetic biological module consisting of only CDT-1 and GH1-1. This strain could ferment cellobiose, but is still inferior to consumption of extracellular glucose in terms of rate and results in a prolonged lag phase [7]. The addition of a functional xylose metabolic pathway resulted in a strain that was able to co-ferment xylose and cellobiose [8]. Further efforts to increase the rate of cellobiose fermentation have included combinatorial transcriptional engineering [9], experimental evolution [10–14], exploration and optimization of cellodextrin transporters [15, 16], and the manipulation of transcription factors [7]. Despite these engineering efforts, cellobiose fermentation by *S. cerevisiae* is still very limited. In contrast, cellulolytic fungi such as *M. thermophila* naturally use cellobiose efficiently.

Thermophilic fungi are excellent producers of thermostable enzymes for industrial applications and can potentially be developed into cell factories to produce chemicals and materials at elevated temperatures. *M. thermophila* is a thermophilic filamentous fungus that can produce a variety of carbohydrate-active enzymes (CAZymes) involved in the degradation of a wide range of biomass sources [17, 18]. Such enzymes can potentially be used in the production of biofuels and chemicals. The fermentation characteristics of *M. thermophila* are suitable for large-scale production [19]. In addition, the availability of its genomic sequence [17] and well-developed genetic tools [20–22] allow for targeted genetic engineering of this thermophilic fungus for the production of biochemicals. Recently, *M. thermophila* was engineered to produce fumaric acid efficiently from glucose [23] and C4-dicarboxylic acids from cellulose and corn cob [24], indicating that *M. thermophila* is a promising system for chemical and fuel production.

Here, we evaluated the transcriptional response of *M. thermophila* to cellobiose during fermentation and constructed strains with enhanced ethanol production. Increasing the sugar-uptake capability significantly increased ethanol production from cellobiose by the fungus to almost 10 g/L, approximately three times that produced by the wild-type (WT) strain. A transcriptomic analysis revealed large-scale transcriptional changes in the engineered efficient cellobiose-utilizing strain, with reduced expression of genes involved in oxidation–reduction reactions and the stress response pathway, and increased expression of genes involved in amino acid biosynthesis. Our results show that the cellulolytic fungus *M. thermophila* is a promising new platform for bioethanol production from cellulosic materials, especially after further metabolic engineering using a consolidated bioprocessing (CBP) strategy to produce strains with high cellulase activity and ethanol fermentation rates.

## Results

### Evaluation of ethanol tolerance of *M. thermophila*

Ethanol tolerance is an important consideration when selecting a host for ethanol production, because it is a critical factor affecting the ability of the microorganism to generate economically viable quantities of ethanol. To assess the feasibility of using the thermophilic fungus *M. thermophila* as an ethanol production system, its ethanol tolerance was determined by inoculating either spores or pre-grown mycelia into fermentation medium containing 7.5% glucose and different concentrations of ethanol and then determining the dry cell weight (DCW) after a defined culture period.

**Fig. 1:**
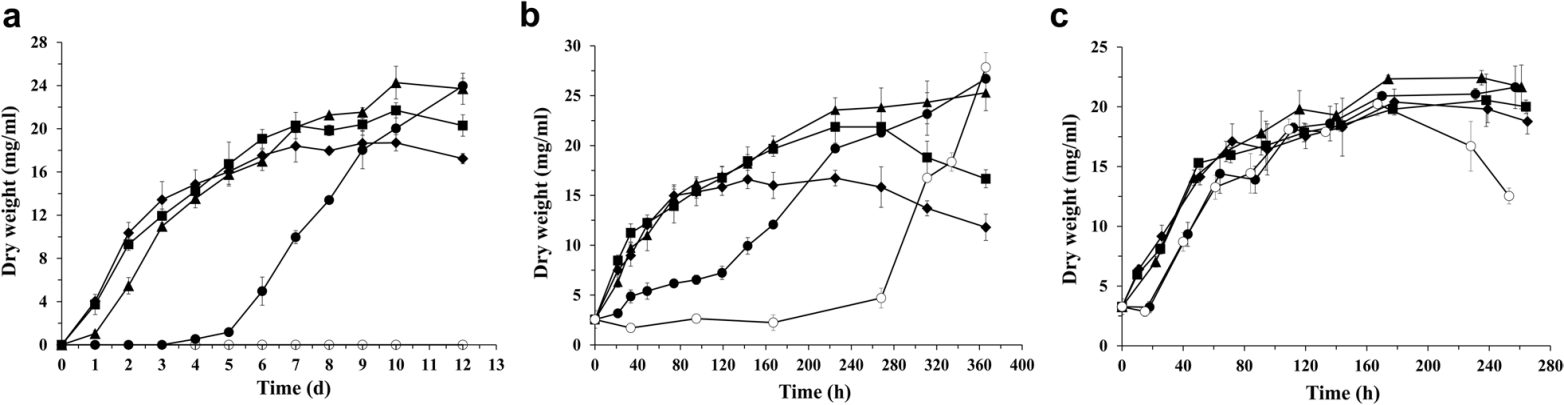
Determination of ethanol tolerance of *M. thermophila*. **a** Spores were inoculated into fermentation medium (2.5 × 10^5^/mL) containing different concentration of ethanol. **b** Spores were inoculated into fermentation medium (2.5 × 10^5^/mL) and grown for 20 h, and then mycelia were transferred to fresh fermentation medium containing different concentration of ethanol. Ethanol concentration used were 0 g/L (closed diamond), 10 g/L (closed square), 20 g/L (closed triangle, 30 g/L (closed circle) and 40 g/L (open circle). **c** Spores were inoculated into fermentation medium (2.5 × 10^5^/ml) and grown for 24 h. Ethanol was added to a final concentration of 50 g/L. After incubation for 1 h (closed diamond), 2 h (closed square), 5 h (closed triangle), 9 h (closed circle) and 12 h (open circle), mycelia were transferred to fresh fermentation medium. Culture conditions: 48 °C, shaking at 150 rpm. Dry cell weight (DCW) was determined at end of culture period. Error bars represent SD from three replicates

When spores were used as the inoculum, mycelia could grow normally in media containing 10 g/L and 20 g/L ethanol with no observed defects. Mycelia grew moderately in medium containing 30 g/L ethanol, but could not grow in medium containing 40 g/L ethanol (Fig. 1a).

The ethanol tolerance of pre-grown mycelia was also determined. As shown in Fig. 1b, mycelia could re-grow in medium containing 40 g/L ethanol, but there was a lag phase. Mycelia did not grow in medium containing 50 g/L ethanol, but were still viable after 12 h in that medium (Fig. 1c), suggesting that *M. thermophila* can tolerate up to 50 g/L ethanol. This is a relatively high level of ethanol tolerance.

At the end of the culture period, the DCW was higher in media containing 10–30 g/L ethanol than in medium containing no ethanol, and higher in media containing 20–30 g/L ethanol than in medium containing 10 g/L ethanol (Fig. 1a, b). These results indicate that *M. thermophila* is able to use ethanol as a carbon source after glucose is depleted.

### Comparison of transcriptomes of *M. thermophila* WT between cellobiose vs. d-glucose as carbon source

*Myceliophthora thermophila* utilizes cellobiose as fast as glucose. There are certain advantages of engineering *M. thermophila* to produce ethanol from cellobiose, a non-repressing sugar, rather than from glucose. To gain further insights into cellobiose utilization by this thermophilic fungus, we compared the transcriptional responses of the *M. thermophila* WT strain between cultures with cellobiose and glucose as the carbon source. The transcriptomes of *M. thermophila* growing in media containing glucose and cellobiose were prepared by the RNA-sequencing technique. Clean reads were mapped to the *M. thermophila* genome sequence and reads per kilobase of exon model per million mapped reads (RPKMs) were calculated as the normalized expression values of each annotated gene. Differential expression analysis was conducted using DESeq [25] (Additional file 1: Table S2). Pearson correlation analysis demonstrated that the biological replicates were reliable for all tested samples (Additional file 1: Table S3).

**Fig. 2:**
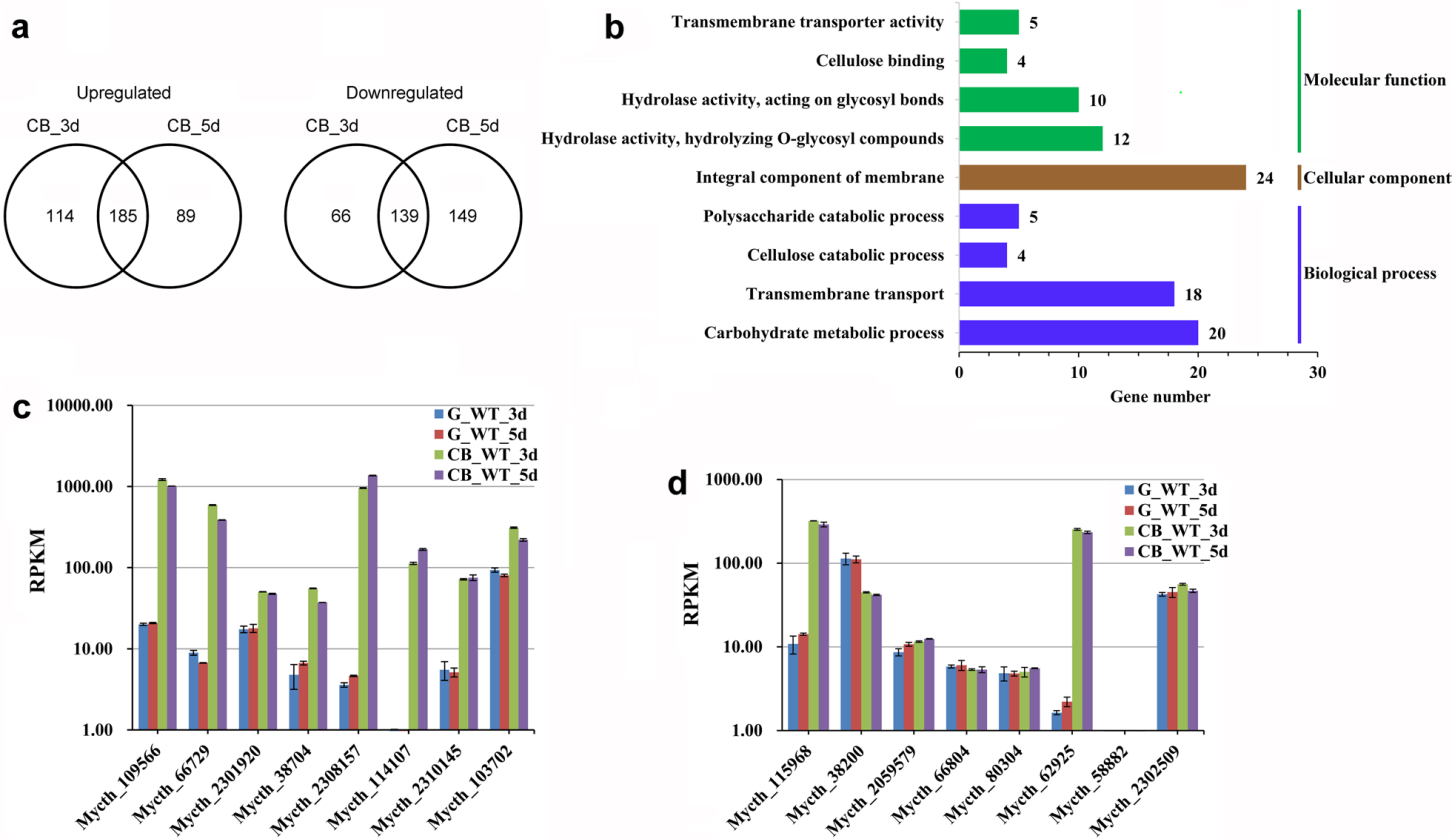
Genes with statistically significant differences in transcript levels between wild-type (WT) *M. thermophila* grown with cellobiose and WT grown with glucose as carbon source. **a** Venn diagram showing upregulated and downregulated genes in WT cells grown with cellobiose after 3 days and 5 days of culture, respectively. **b** Gene Ontology analysis of 185 commonly upregulated genes in **a**. **c** Transcript levels of selected upregulated genes. **d** Transcript levels of all genes encoding glucosidases

Compared with d-glucose as the carbon source, cellobiose resulted in the significant differential expression of 742 genes at 3-day and 5-day fermentation, of which 388 genes were upregulated and 354 genes were downregulated. There were 185 upregulated genes and 139 downregulated genes common to the 3-day and 5-day cultures, respectively (Fig. 2a). Gene ontology (GO) analysis showed that, among the 185 upregulated genes, those involved in cellulose degradation were the most enriched category (Fig. 2b and Additional file 1: Table S4). This category included the following 15 cellulase genes: Mycth_109566 (orthologous to *cbh*-*1* NCU07340), Mycth_66729 (orthologous to *cbh*-*2* NCU09680), Mycth_115968 (*β*-glucosidase, orthologous to *gh1*-*1* NCU00130), Mycth_62925 (*β*-glucosidase, orthologous to *gh3*-*6* NCU07487), Mycth_83041 (orthologous to *gh76*-*3* NCU08127), Mycth_110651 (orthologous to *gh61*-*7* NCU00836), and two hemicellulase genes (*β*-xylosidase Mycth_80104, orthologous to *gh43*-*5* NCU09652 and endoxylanase Mycth_116553, orthologous to *gh10*-*2* NCU08189). Compared with glucose as the carbon source, cellobiose resulted in upregulated expression of four transcription factors that regulate cellulase expression (Mycth_38704 orthologous to *clr*-*2* NCU08042, Mycth_2305813 orthologous to *clr*-*3* NCU05846, Mycth_2301920 orthologous to *col*-*26* NCU07788, and Mycth_2310145 orthologous to *xlr*-*1* NCU06971), two genes encoding cellodextrin transporters (Mycth_114107, orthologous to *cdt*-*2* NCU08114 and Mycth_84164, orthologous to *clp1* NCU05853), one gene encoding a high-affinity glucose transporter (Mycth_2308157 orthologous to *hgt*-*1* NCU10021) (Fig. 2c). Mycth_38704 (*clr*-*2*), Mycth_2305813 (*clr*-*3*), Mycth_2310145 (*xlr*-*1*), Mycth_114107 (*cdt*-*2*), and Mycth_84164 (*clp1*) are components of the ‘‘Mt Avicel regulon” [26] and their orthologs in *N. crassa* are components of the “Nc Avicel regulon” [27]. CLR-2, CLR-3, XLR-1, CDT-2, and CLP1 are known to play essential roles in (hemi)-cellulose utilization by *N. crassa* [27–31]. Mycth_2301920, which was significantly induced by cellobiose as the carbon source, is the *M. thermophila* ortholog of the Zn(II)2Cys6 transcription factors AmyR in *Aspergillus* and COL-26 in *N. crassa,* which are essential for starch utilization [32–34].

Interestingly, a gene encoding aldose 1-epimerase (AEP, Mycth_103702 orthologous to NCU08398), which catalyzes the interconversion between *β*-form and *α*-form sugars, was significantly upregulated in *M. thermophila* cultured with cellobiose, compared with that cultured with glucose. Previously, this gene was significantly upregulated when plant biomass was used as the carbon source [35]. Similarly, in *N. crassa*, the gene encoding AEP (NCU08398) was significantly induced by biomass and Avicel [27, 36, 37], indicating that high AEP expression may allow cellulolytic fungi to utilize cellobiose as well as plant biomass. Interestingly, the deletion of *GAL10* (encoding aldose 1-epimerase) from the engineered cellobiose-utilizing *S. cerevisiae* expressing CDT-1 and GH1-1 led to the complete loss of cell growth on cellobiose [38]. To investigate the role of AEP in cellobiose utilization of *M. thermophila*, we constructed the *aep* disruption mutant (Δ*aep*) by CRISPR/Cas9 system (Additional file 2: Fig. S1a). Four mutants were randomly selected for growth assay. There were no differences of growth on glucose or cellobiose between Δ*aep* mutants and WT strain (Additional file 2: Fig. S1b). When cultured in fermentation media containing 7.5% glucose or cellobiose, the ethanol production of Δ*aep* mutants was reduced by 75% and 43%, respectively (Additional file 2: Fig. S1c). The biomass of Δ*aep* mutants was not reduced, even better when glucose was used (Additional file 2: Fig. S1d). These results implicated the functional discrepancy of AEP among species.

In *N. crassa*, cellobiose induced the expression of lignocellulolytic genes in an engineered strain with three deleted *β*-glucosidase genes, but not in the WT strain [39]. Of the 185 upregulated genes in *M. thermophila* cultured with cellobiose as the carbon source, 36 encoded components of the ‘‘Mt Avicel regulon”. Seven of those genes were among 38 genes showing increased expression levels under starvation conditions in another study [34]. Three of the seven genes (Mycth_66729, Mycth_115968, and Mycth_103702) showed similar moderate induction with cellobiose as the carbon source and under starvation conditions. The transcript levels of the other four genes (Mycth_110651, Mycth_84164, Mycth_114107 and Mycth_5342) were much higher on cellobiose medium (Additional file 1: Table S4) [34]. These data indicate that the induction of these 185 genes in *M. thermophila* by cellobiose is due to a combination of relief from CCR and the presence of an inducer (cellobiose), like the response of *N. crassa* to xylan as the carbon source [29].

Of the eight genes encoding *β*-glucosidases, only Mycth_115968 and Mycth_62925 with no predicted secretory signal peptides were induced by cellobiose (Fig. 2d). In previous studies, two genes encoding intracellular *β*-glucosidases were also induced by Avicel [26] and plant biomass [35], indicating that they are the most relevant enzymes for converting cellobiose to glucose when *M. thermophila* is cultured with either cellobiose or plant-derived biomass as the sole carbon source.

Of the 139 downregulated genes in *M. thermophila* cultured with cellobiose as the carbon source, those involved in ammonium transport, nitrogen metabolism, and membranes were the most enriched (Additional file 1: Table S5), suggesting a probable correlation between carbon and nitrogen metabolism in *M. thermophila*, as is the case in *N. crassa* [33].

Cellobiose can also be cleaved to glucose-1-phosphate and glucose by cellobiose phosphorylase. The genome of *M. thermophila* contains only one predicted gene (Mycth_2308030, *Mtcpp*) encoding cellobiose phosphorylase, orthologous to *ndvB* (NCU09425) from *N. crassa* which encodes a cellobionate phosphorylase. *ndvB* was shown to be involved in cellobionate and cellobiose utilization in *N. crassa* [40, 41]. Recently, the role of MtCPP was investigated and showed that MtCPP is indeed the functional component of cellobiose phosphorolytic pathway [42]. However, the transcript levels of *Mtcpp* were not induced by cellobiose in *M. thermophila* WT strain (Additional file 1: Table S2) but in an engineered malate-producing strain JG207 [42].

The differentially expressed genes in *M. thermophila* between cellobiose and glucose as the carbon source included a few genes involved in central carbon metabolism pathways. To explore this in more detail, the transcript levels of three hexokinase genes (Mycth_2297364, Mycth_2295756 and Mycth_2306777) were analyzed. Compared with cells grown with glucose as the carbon source, those grown with cellobiose showed increased transcript levels of Mycth_2297364 (by 3.1- and 6.8-fold at 3 day and 5 day, respectively) but no difference in the transcript levels of Mycth_2295756 and Mycth_2306777 (Additional file 1: Table S2). Similarly, *Hxk1* of *S. cerevisiae* was highly transcribed in cellobiose-grown cells [7]. Mycth_2297364 also showed elevated transcript levels in cells grown with glucose, starch, and Avicel compared with cells grown in starvation conditions [26, 34]. These data indicate that Mycth_2297364 is the predominant isoenzyme when cells are grown with fermentable carbon sources. Its elevated expression in cells cultured with cellobiose as the carbon source may contribute to efficient utilization of this substrate. Other genes in central carbon metabolic pathways showing significant differential expression between glucose and cellobiose conditions included those encoding a trehalose-6-phosphate synthase (Mycth_2309547), a glycogen phosphorylase (Mycth_2309843), and an isocitrate lyase (Mycth_110871).

### Engineering of *M. thermophila* to produce ethanol from cellobiose

The genome of *M. thermophila* contains genes encoding a pyruvate decarboxylase (Mycth_112121) and a number of alcohol dehydrogenases, indicating that ethanol can be produced from pyruvate. In standard fermentation medium, the *M. thermophila* WT strain produced around 3 g/L ethanol with glucose or cellobiose as the carbon source. This confirmed that the ethanol production pathway is functional in this thermophilic fungus, which encouraged us to engineer this cellulolytic fungus as a new ethanol producer.

**Fig. 3:**
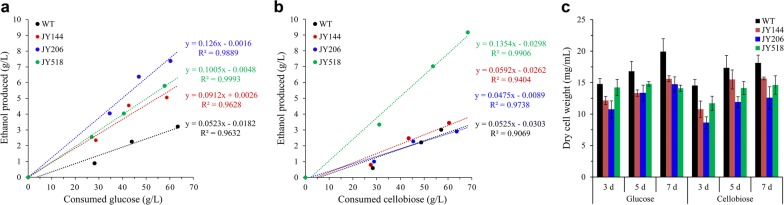
Profiles of ethanol produced and sugar consumed by *M. thermophila* strains grown with glucose (**a)** and cellobiose (**b**) as carbon source. Biomass of corresponding strains is shown in **c**. Strains were cultured at 48 °C with shaking at 150 rpm. Error bars represent SD from three replicates

**Fig. 4:**
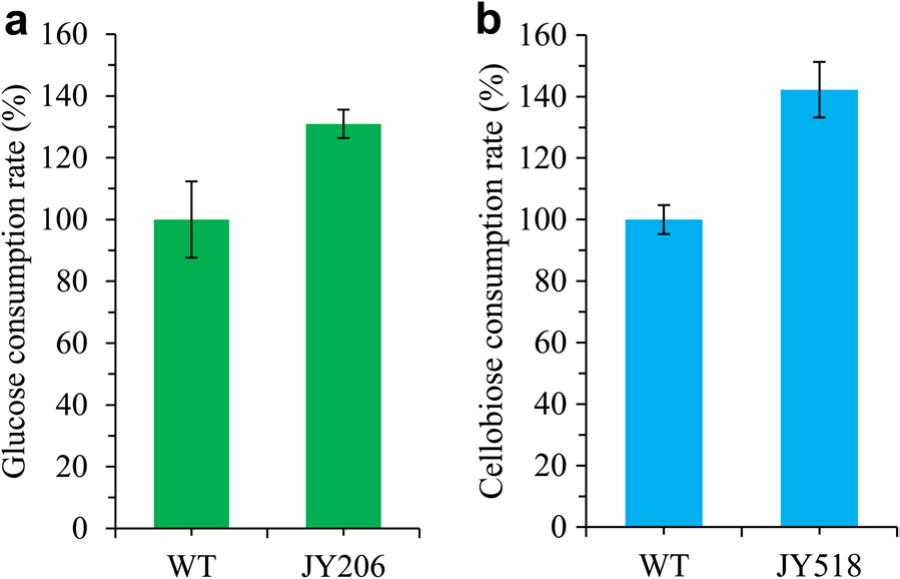
Assay of sugar consumption rate for glucose (**a**) and cellobiose (**b**)

We speculated that its glycolytic flux is not fast enough to drive ethanol production efficiently. In *S. cerevisiae*, if upper glycolysis (upstream of fructose 1,6-bisphosphate) outpaces lower glycolysis (downstream of fructose 1,6-bisphosphate), an imbalanced state with reduced ATP production occurs [43]. Therefore, we first introduced *S. cerevisiae Adh1* (*ScAdh1*, without codon optimized), which encodes an alcohol dehydrogenase (ADH) that catalyzes the conversion of acetaldehyde into ethanol, into the *M. thermophila* WT strain to accelerate lower glycolysis. The copy number of the chromosomally integrated *ScAdh1* in the transformants was determined by real-time quantitative PCR. Strain JY144, which produced the highest titer of ethanol among the transformants (Additional file 3: Fig. S2a), harbored 3 copies of *ScAdh1* (Additional file 3: Fig. S2c) and was chosen for further engineering. JY144 produced 5.06 g/L ethanol from glucose, representing a 57% increase compared with the WT strain (Fig. 3a). The alcohol dehydrogenase activity of JY144 strain was 40% higher than that of WT strain (0.78 ± 0.06 U/mg vs. 0.55 ± 0.08 U/mg), indicating that the enhanced ADH activity contributed to the increased ethanol production in JY144. We further introduced the *N. crassa* low-affinity glucose transporter GLT-1 (NCU01633, without codon optimized) into JY144 to enhance glucose transport. The resulting strain JY206 (produced the highest titer of ethanol among the transformants with 4 copies of *glt*-*1* (Additional file 3: Fig. S2b, d) showed improved ethanol production (7.4 g/L from glucose) compared with 3.2 g/L produced by the WT strain (Fig. 3a). The glucose consumption rate of JY206 increased by 31% compared to WT strain (Fig. 4a). Surprisingly, when cellobiose was supplied as the carbon source, JY144 and JY206 performed no better than WT in terms of ethanol production (Fig. 3b), suggesting that some other engineering strategies should be considered for cellobiose fermentation.

**Fig. 5:**
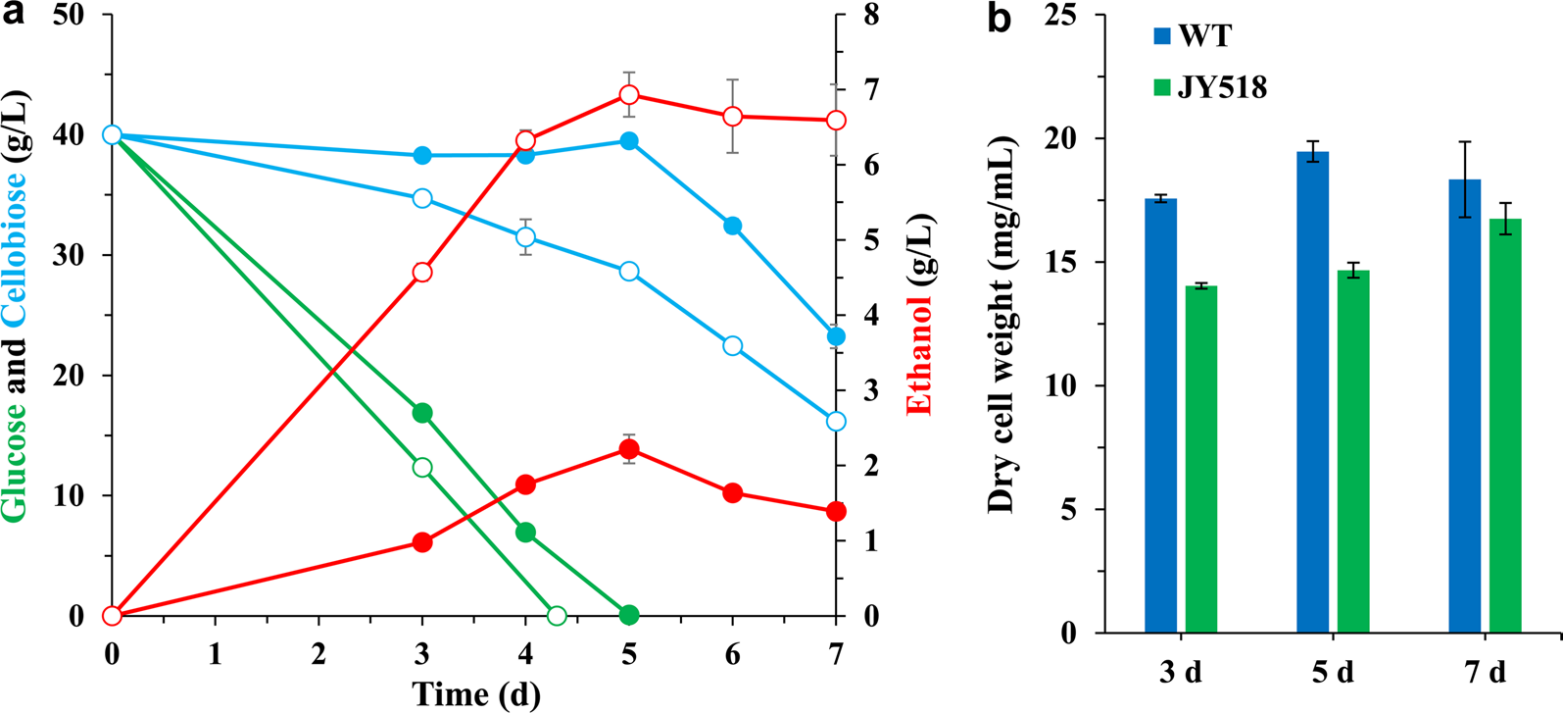
Co-fermentation of glucose and cellobiose by WT (closed circle) and JY518 strain (open circle). **a** Fermentation profiles during the fermentation of sugar mixtures containing 40 g/L of glucose and 40 g/L of cellobiose. **b** Dry weights of mycelia in the culture during fermentation

We speculated that increasing the intracellular cellobiose concentration might increase ethanol production if the native cellodextrin transporters do not take up enough cellobiose for strong glycolytic flux towards ethanol production. Since cellobiose consumption was found to be strongly correlated with ethanol production rates in cellobiose-utilizing *S. cerevisiae* (Ha et al. [10]), we introduced the cellodextrin transporters CDT-1/-2 without codon optimized from the filamentous fungus *N. crassa* into JY144 strain. CDT-1/-2 can work heterologously in *S. cerevisiae*, so we introduced them into two lactate dehydrogenase gene sites, Mycth_38939 and Mycth_110317, respectively, in *M. thermophila*. This resulted in the addition of two new cellodextrin transporters as well as the disruption of a potential lactate production pathway. The resulting strain JY518 showed significantly improved ethanol production from cellobiose (9.2 g/L, approximately three times that of the WT strain) (Fig. 3b). The cellobiose consumption rate of JY518 increased by 42% compared to WT strain (Fig. 4b). When glucose was used as the carbon source, the ethanol production of JY518 was no different from that of JY144, maybe because CDT-1/-2 do not have glucose uptake activity. The correlation between the sugar consumption and ethanol production was observed in the tested strains (Fig. 3a, b). The engineered strains converted more carbon flux towards ethanol production which was more obvious if the biomass was taken into consideration, since the less biomass was observed in strains with higher ethanol production (Fig. 3c). Co-consumption of glucose and cellobiose by JY518 strain was also investigated. Compared to WT strain which only utilized cellobiose after glucose was depleted, JY518 was capable of co-utilizing these two sugars and also producing more ethanol than WT strain (6.9 ± 0.3 g/L vs. 2.2 ± 0.2 g/L) (Fig. 5a).

All the engineered strains showed reduced biomass compared with that of WT, regardless of whether the carbon source was glucose or/and cellobiose (Figs. 3c, 5b). In *S. cerevisiae,* overexpression of most glycolytic proteins was found to cause growth defects [44]. We speculated that overexpression of heterologous proteins in the *M. thermophila* strains negatively affected their growth. These results indicate that enhancing sugar uptake is a useful approach to increase the glycolytic flux to ethanol production, despite the adverse effects of heterologous protein overexpression on growth.

### Comparative transcriptional analysis of WT and JY518 with cellobiose as carbon source

**Fig. 6:**
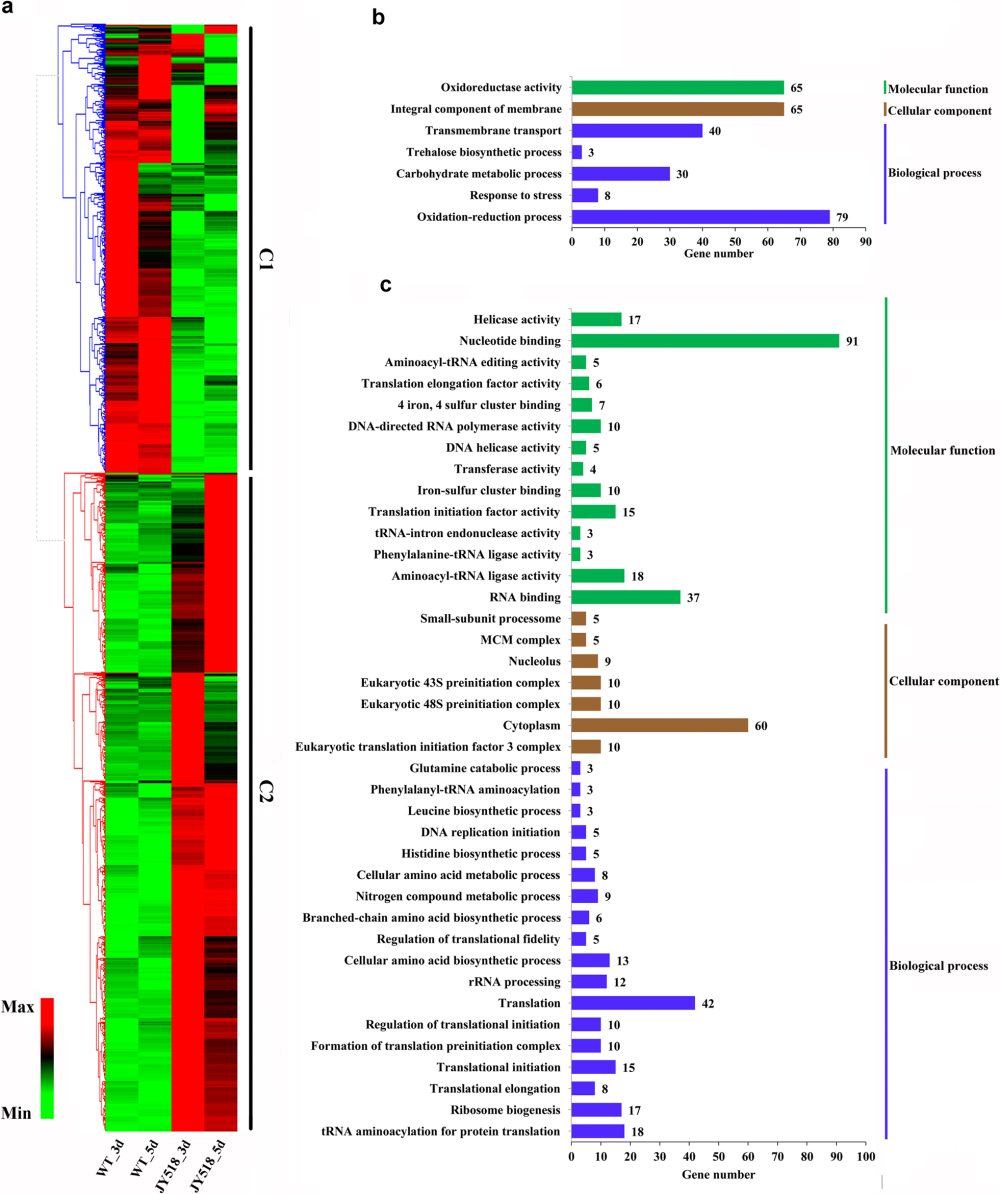
Genes with statistically significant differences in transcript levels between JY518 and wild-type (WT) strains of *M. thermophila* cultured with cellobiose as carbon source. **a** Hierarchical clustering of RPKM values for differentially expressed genes. **b** and **c** Gene Ontology analysis of downregulated genes and upregulated genes (C1 and C2 cluster, respectively)

To investigate the gene expression profile of the effective ethanol production strain JY518 during cellobiose utilization, we compared its transcriptome with that of the WT strain with cellobiose as the carbon source. We found that 1051 genes were upregulated and 710 genes were downregulated at either 3 day or 5 day of culture in JY518 (Fig. 6a and Additional file 1: Table S2). Among the 710 downregulated genes, those involved in oxidation–reduction reactions, integral membrane components, transmembrane transport, response to stress, and carbohydrate metabolism were the most enriched (Fig. 6b and Additional file 1: Table S6). Among the downregulated genes was a group of genes encoding various products with a FAD-binding domain, such as Mycth_39133, Mycth_2140533, Mycth_2299483, Mycth_73194, Mycth_2297213, Mycth_109870 and Mycth_70573. Both Mycth_2140533 and Mycth_109870 are orthologous to *sol5* gene of *Alternaria solani* encoding solanapyrone synthase involved in secondary metabolite solanapyrone A biosynthesis [45]. Mycth_73194 is orthologous to *pvdA* of *Pseudomonas aeruginosa* encoding l-ornithine N(5)-oxygenase involved in pyoverdine siderophore biosynthesis [46]. Mycth_39133 encodes a putative D-aspartate oxidase, Mycth_2297213 encodes a putative ferric-chelate reductase and Mycth_70573 encodes a putative pyridine nucleotide-disulphide oxidoreductase. This indicates the secondary metabolite pathways such as solanapyrone synthesis were weakened in engineered ethanol production strain on cellobiose. However, the exactly connections between secondary metabolism and ethanol production in engineered strain grown on cellobiose is not known now. The downregulated genes also included stress response-related genes (GO: 0006950) including Mycth_2133483, Mycth_2306260, and Mycth_80427. Mycth_2133483 is an ortholog of the *S. cerevisiae* ATP-dependent Hsp90 family molecular chaperones Hsc82 and Hsp82, which assist in the maturation of a large set of target proteins. Mycth_2306260 is an ortholog of *S. cerevisiae* Aha1, a co-chaperone that binds to Hsp82p and activates its ATPase activity [47]. In *S. cerevisiae*, ATP hydrolysis by Hsp90 is required to maintain its function [48, 49]. Mycth_80427 encodes a heat shock protein orthologous to *S. cerevisiae* Hsp104. During heat shock, Hsp104 contributes to the simultaneous increase in both the accumulation and degradation of trehalose in *S. cerevisiae* [50]. Trehalose plays a vital role in the heat shock response of *S. cerevisiae* and *M. thermophila* [51, 52]. Consistent with the downregulation of Mycth_80427 (*hsp104*), genes involved in trehalose biosynthesis (Mycth_2082262, Mycth_2295490, Mycth_2309547) were also downregulated in JY518 compared with WT grown with cellobiose as the carbon source (Fig. 6b and Additional file 1: Table S2). These results suggest that the heat stress response is probably impaired in JY518. Indeed, JY518 grew better than WT strain on cellobiose plates at elevated temperature but more sensitive to H_2_O_2_-induced oxidative stress (Additional file 4: Fig. S3a). The impaired response of JY518 strain to heat stress was more obvious when cultivated in fermentation media containing cellobiose at 55 °C, since the better growth was observed in JY518 compared to WT strain (Additional file 4: Fig. S3b).

Genes involved in transcription, translation, and amino acid biosynthesis were the most enriched among the 1051 upregulated genes in JY518 compared with WT grown with cellobiose as the carbon source (Fig. 6c and Additional file 1: Table S7). In engineered cellobiose-utilizing *S. cerevisiae,* cellobiose induces mitochondrial activation and reduces amino acid biosynthesis, compared with glucose as the substrate [7]. Our JY518 strain consumed more cellobiose and produced more ethanol than did the WT strain because of the faster glycolytic flux conferred by CDT-1/-2 (Fig. 3b). In JY518, genes involved in amino acid biosynthesis were induced (Fig. 6c). These results indicate a correlation between glycolytic flux and amino acid biosynthesis. Since WT *M. thermophila* showed comparable sugar consumption and ethanol production rates between glucose and cellobiose as the carbon source (Fig. 3a, b), glycolytic flux should be similar on both substrates in the WT. Consistently, the transcript levels of genes involved in amino acid biosynthesis showed no significant differences in WT between cellobiose and glucose as the carbon source (Additional file 1: Table S2). We speculated that the enhanced amino acid biosynthesis and translation in JY518 led to enhanced biosynthesis of enzymes involved in glycolysis, resulting in greater ethanol production from cellobiose in this engineered strain.

**Fig. 7:**
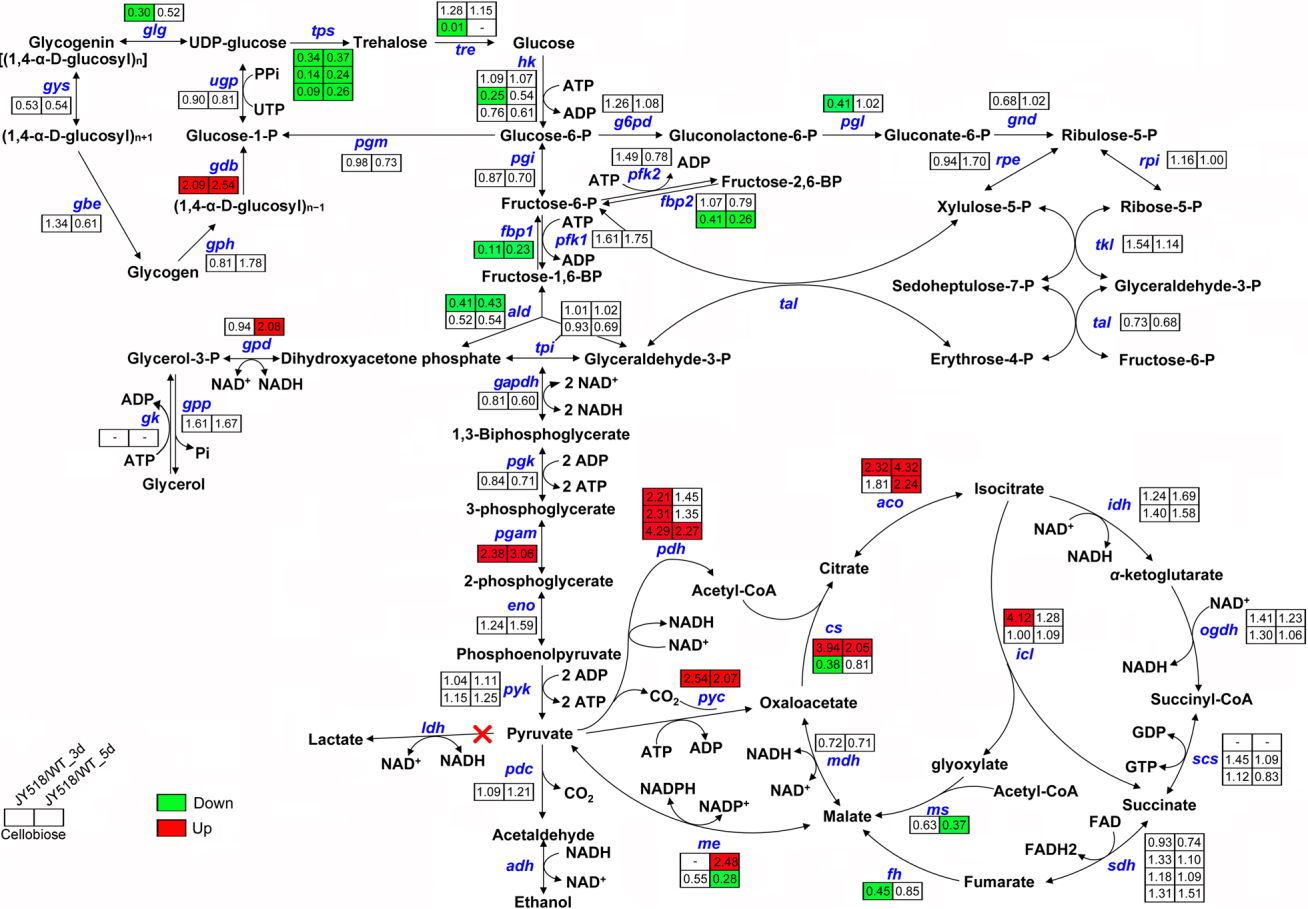
Expression of genes in central metabolic pathway in wild-type (WT) and engineered *M. thermophila* strain JY518. Numbers represent RPKM ratio of JY518 strain to WT strain. Upregulated genes (ratio > 2, *P* value < 0.05) are shown in red; downregulated genes (ratio < 0.5, *P* value < 0.05) are shown in green. -, RPKM < 20 both in WT and JY518 strain at 3 day and 5 day of culture. Detailed data are shown in Additional file 1: Table S8

**Fig. 8:**
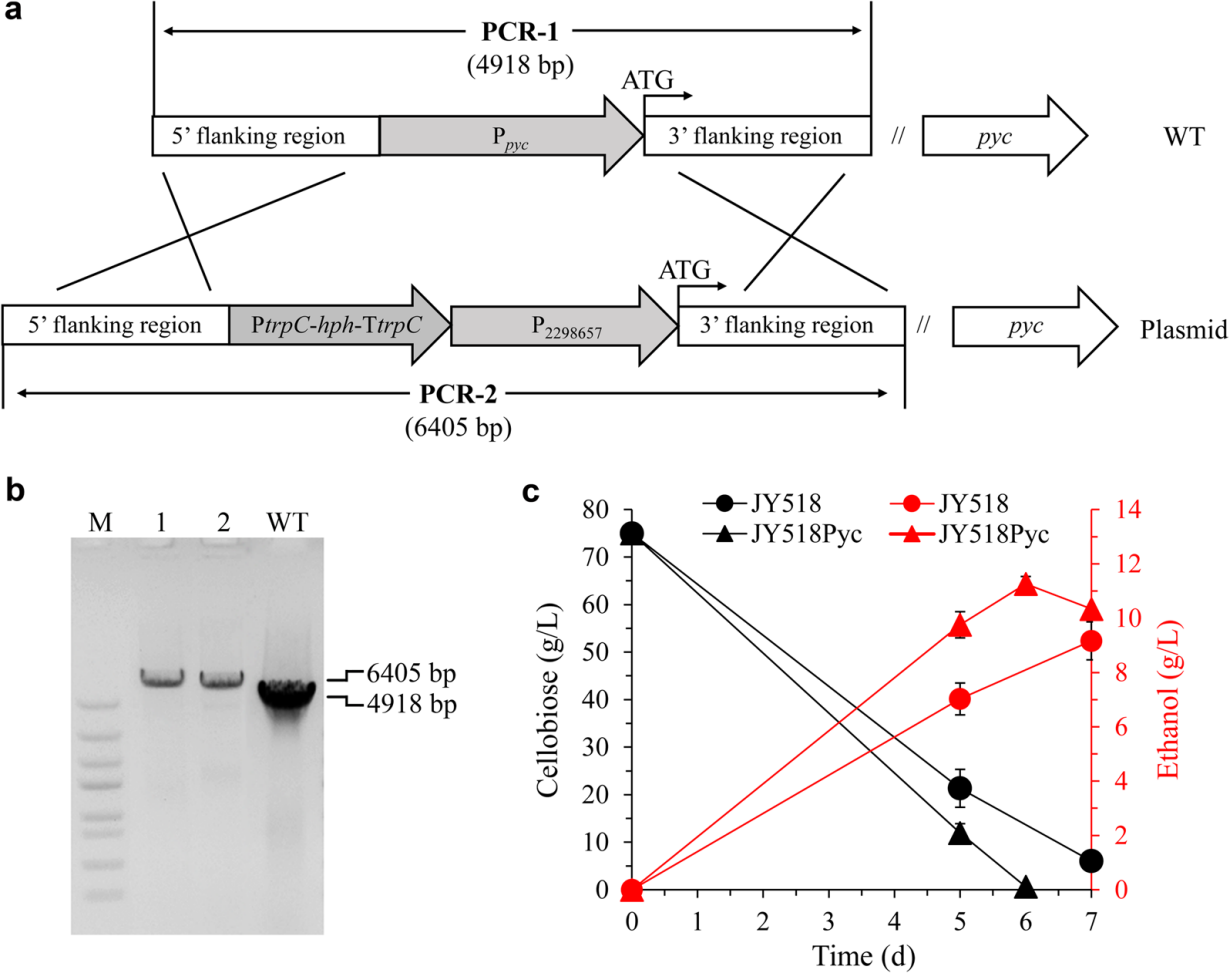
Construction and characterization of JY518Pyc strain. **a** Strategy for construction of JY518Pyc via homologous recombination. **b** Confirmation of JY518Pyc by PCR. PCR1 and PCR2 were performed with the primers 2295692QC-F/2295692QC-R. The PCR product of JY518Pyc is 6.4 kb (Lane 1 and 2) while that of WT is 4.9 kb. **c** Fermentation profile of JY518Pyc strain

Next, we focused on genes involved in central metabolism. *fbp1,* whose product catalyzes the conversion of fructose-1,6-bisphosphate (FBP) to fructose-6-phosphate, was significantly downregulated and *pgam* encoding phosphoglycerate mutase converting 3-phosphoglycerate into 2-phosphoglycerate was upregulated, indicating that glycolytic flux was indeed accelerated in JY518. The *gpd* gene in glycerol production pathway was upregulated at 5-day culture (Fig. 7). Consistently, the glycerol production of JY518 was higher than that of WT strain both at 5-day and 7-day cultivation (Additional file 5: Fig. S4). The *pyc* gene, whose products catalyze the conversion of pyruvate to oxaloacetate, was also upregulated in JY518 (Fig. 7). Disruption of *pyc* may redirect more carbon flux into ethanol production. We failed to obtain *pyc* disruption mutant using CRISPR–Cas9 system, instead, we replaced *pyc* native promoter with a constitutive low-expression promoter p2298657 (RPKM < 20) to alleviate its expression in JY518 strain. Resulting strain JY518Pyc (Fig. 8a, b) increased ethanol production by 23% compared with JY518, reach to 11.3 g/L, and the cellobiose utilization also got improved (Fig. 8c). The *pdh* gene whose product converts pyruvate to acetyl-CoA was also upregulated in JY518 strain, it would be a good target to engineer in the future. Finally, consistent with the increased transcript levels of *pyc* and *pdh*, most genes involved in amino acids biosynthesis whose precursor is pyruvate or *α*-ketoglutarate were also upregulated in JY518 strain, especially at 5-day fermentation (Additional file 6: Fig. S5).

## Discussion

Thermophilic fungi are promising resources of thermostable enzymes for industrial applications with stability at 70–80 °C [53]. *M. thermophila* is capable of degrading various plant-derived biomasses [17, 35] and is a promising biocatalyst for the production of biochemicals [23, 24]. Besides, its high-temperature fermentation at 45–50 °C can significantly reduce cooling costs and susceptibility to contamination. If developed successfully, the use of *M. thermophila* to produce ethanol directly from plant biomass would be a very attractive system of fungal CBP. However, its ethanol production from Avicel (cellulose) is very low, less than 0.1 g/L in the WT *M. thermophila* strain (data not shown). Thus, targeted engineering work for fine-tuning the cellulase expression and better fermentative performance is required to construct a fungal CBP strain. Our study represents the start of such an engineering process. The engineered strain produced 11.3 g/L ethanol, three times increased compared with the WT strain, from cellobiose, a major component of cellulose hydrolysates.

*Saccharomyces cerevisiae* can ferment sugars even in the presence of oxygen and at high glucose concentrations, a phenomenon referred to as the Crabtree effect [54]. As demonstrated in this study, 11.3 g/L ethanol was produced by *M. thermophila* under non-oxygen-limited conditions, suggesting that this fungus might have the capability to be engineered as a Crabtree-positive microbe to produce ethanol well by thoroughly metabolic reconstruction. Unlike *S. cerevisiae*, *M. thermophila* cannot grow under anaerobic conditions. Its growth under semi-aerobic conditions (when the vessel was sealed with a silicon plug stopper) was significantly impaired (Additional file 7: Fig. S6a), due to the low DO level in the medium (Additional file: 7: Fig. S6b).

In *S. cerevisiae*, the balance between respiration and fermentation is the main factor affecting sugar utilization and ethanol production under aerobic conditions [7, 55, 56]. Our transcriptomic analysis of engineered *M. thermophila* showed that enhanced cellobiose utilization affects oxidation–reduction processes (Fig. 6b) as well as growth and stress responses. Therefore, manipulating certain oxidation–reduction processes might improve the efficiency of ethanol production. For example, overexpression of NADH oxidase to maintain the redox balance [57], deletion of ADH that involved in ethanol utilization [58] or deletion of mitochondrial NADH dehydrogenases to shift from respiratory to fermentative metabolism [59]. Further research is required to test these ideas experimentally. In cellobiose-utilizing *S. cerevisiae*, limited activity of Pfk1 (which catalyzes the phosphorylation of fructose-6-phosphate) was found to be a major bottleneck in cellobiose consumption [60]. Identification of rate-limiting step(s) in glycolytic pathway during cellobiose utilization would facilitate the engineering of *M. thermophila* to produce biochemicals. Multiple metabolic engineering strategies including enhancing cellobiose uptake to enhance the speed of glycolytic pathway have shown to be effective for final bio-based chemicals production in our previously engineered hyper malic acid-producing strain [24, 42]. Therefore, further increase of the glycolytic flux in combination with downregulated flux towards TCA cycle could be a good approach to increase more flux towards ethanol. Further metabolomic research needs to be employed to understand the underlying regulatory network behind biological responses at the mechanistic level.

## Conclusions

The results of our study show that *M. thermophila* is a promising system for ethanol production from the biomass-derived sugar, cellobiose. Engineered strain showed three times increased ethanol production rates. This study demonstrates how a thermophilic fungus can be engineered to produce ethanol or other fuels directly from lignocellulosic substrates by fungal CBP technology.

## Methods

### Strains, media, and growth conditions

The *M. thermophila* strains used in this study are listed in Table 1. For sporulation, *M. thermophila* strains were grown on Vogel’s minimal medium supplemented with 2% glucose (MM) or xylose at 37 °C for 10–15 days. For flask culture, conidia of *M. thermophila* were inoculated into 100 mL fermentation medium to a final concentration of 2 × 10^5^ conidia/mL in a 250-mL Erlenmeyer flask. The fermentation medium (per liter) consisted of 75 g glucose or cellobiose, 10 g yeast extract, 0.15 g KH_2_PO_4_, 0.15 g K_2_HPO_4_, 0.1 g MgSO_4_·7H_2_O, 0.1 g CaCl_2_·2H_2_O, 1 mL biotin (0.1 g/L), and 1 mL trace elements (Vogel’s salts). *Escherichia coli* DH5*α* was used for plasmid amplification, and was cultured in Luria–Bertani (LB) medium supplemented with antibiotics as necessary.

**Table 1:** Strains used in this study

Strain	Characteristics	Source
*Myceliophthora thermophila* ATCC 42464	Wild-type strain (WT)	ATCC
JY144	WT::P*tef*-*ScAdh1*-T*trpC*	This study
JY206	JY144::P*gpd*-*glt1*-T*cbhI*	This study
JY518	JY144::Pgpd-*cdt1*-T*trpC*/ΔMycth_38939::P*pdc*-*cdt2*-T*chbI*/ΔMycth_110317	This study
Δ*aep*	Mycth_103702 deletion mutant on WT background	This study
JY518Pyc	Native promoter of *pyc* was changed to P2298657 in JY518	This study

*ATCC* American Type Culture Collection

### Construction of expression and deletion plasmids

The primers used in this study are listed in Additional file 1: Table S1. The *Adh1* gene of *S. cerevisiae* was amplified using the primers ScADH1-F/ScADH1-R and assembled into *BamH*I-digested pAN52-MtP*tef*-T*tprC* using the NEB Gibson assembly kit (New England Biolabs, Ipswich, MA, USA). The 5′ and 3′ flanking regions of Mycth_38939 were amplified using the primers 38939-up-F/38939-up-R and 38939-down-F/38939-down-R, respectively. The P*gpd*-*cdt1*-T*trpC* cassette was amplified from pAN52-Mt*gpdA*-*cdt1*-T*tprC* using pAN52-F/TtrpC-bar-R2. The amplified 5′, 3′, and P*gpd*-*cdt1*-T*trpC* fragments were assembled and ligated into *Bgl*II-digested pCAMBIA-0380 using the NEB Gibson assembly kit, yielding pCDT1. The promoter of the gene encoding pyruvate decarboxylase (P*pdc*) was amplified from *M. thermophila* genomic DNA using the primers Ppdc-F/Ppdc-R. The *cdt2*-T*chbI* fragment was amplified from the plasmid containing the *cdt2*-T*cbhI* cassette using the primers CDT2-F/TcbhI-R. P*trpC*-*bar* and T*trpC* were amplified using the primers P-bar-F/P-bar-R and TtrpC-bar-F/TtrpC-bar-R, respectively, and then assembled into *Bgl*II-digested pCAMBIA-0380 to produce p0380-P-*bar*-T. P*trpC*-*bar*-T*trpC* was amplified from p0380-P-*bar*-T using the primers P-bar-F3/TtrpC-bar-R. The P*pdc*, *cdt2*-T*chbI*, and P*trpC*-*bar*-T*trpC* fragments were assembled into *Bgl*II-digested pCAMBIA-0380. The 5′ and 3′ flanking regions of Mycth_110317 were amplified using the primers 110317-up-F/110317-up-R and 110317-down-F/110317-down-R, respectively. The large fragment containing P*pdc*-*cdt2*-T*chbI*-P*trpC*-*bar*-T*trpC* was amplified using Ppdc-F2/TtrpC-bar-R2 and assembled together with 5′ and 3′ fragments of Myth_110317 into *Bgl*II-digested pCAMBIA-0380 to produce pCDT2. For overexpression of *glt1* (NCU01633), the ORF of *glt1* was amplified from cDNA of *N. crassa* using the primers 1633-F/1633-R and Pgpd was amplified with the primers Pgpd-F/Pgpd-R. The fragments *glt1* and P*gpd*, and the T*cbhI*-P*trpC*-*bar*-T*trpC* cassette (amplified from pCDT2 using primers TcbhI-F/TtrpC-bar-R) were assembled into *Bgl*II-digested pCAMBIA-0380.

For *aep* (Mycth_103702) deletion, the 5′ and 3′ flanking regions were amplified using primers 103702-up-F/103702-up-R and 103702-down-F/103702-down-R, respectively. P*trpC*-*bar* was amplified from pPK2BarGFPb [20] using 103702-bar-F/103702-bar-R. The fragment 5′-P*trpC*-*bar*-3′ was created by overlapping PCR and cloned into the pJET1.2/blunt cloning vector. To replace the promoter of *pyc*, the 5′ and 3′ flanking regions of P*pyc* were amplified using primers 2295692-up-F/2295692-up-R and 2295692-down-F/2295692-down-R, respectively. P*trpC*-*hph*-T*trpC* was amplified from pCSN44 (GenBank accession NO. LT726870) using HygR-F/HygR-R. p2298657 was amplified from genomic DNA of *M. thermophila* using P2298657-F/P2298657-R. The fragment 5′-P*trpC*-*hph*-T*trpC*-p2298657-3′ was created by overlapping PCR and cloned into the pJET1.2/blunt cloning vector.

To select for specific sgRNAs targeting *ldh1* (Mycth_38939), *ldh2* (Mycth_110317), *aep* (Mycth_103702) and promoter of *pyc* (Mycth_2295692), we used the sgRNACas9 tool [61] to identify sgRNA target sites with high scores. Then, a target-directed *M. thermophila* U6 promoter-driven sgRNA was created by overlapping PCR and cloned into the pJET1.2/blunt cloning vector, giving the corresponding plasmids U6p-*ldh1*-sgRNA, U6p-*ldh2*-sgRNA, U6p-*aep*-sgRNA and U6p-P*pyc*-sgRNA. The Cas9-expression PCR cassette P*tef1*-*Cas9*-T*tprC* was amplified using Ptef-cas-F/TtprC-cas-R from the plasmid p0380-*bar*-P*tef1*-*Cas9*-T*tprC* [21]. All the constructed plasmids were verified by sequencing.

### Transformation of *M. thermophila*

Protoplasts of *M. thermophila* were transformed as previously described [62]. For gene expression, 10 μg linearized plasmid was used. For gene disruption by the CRISPR/Cas9 system, 10 μg Cas9-expression PCR cassette P*tef1*-*Cas9*-T*tprC* from p0380-*bar*-P*tef1*-*Cas9*-eGFP-T*tprC* [21], gRNA expression PCR cassette, and the corresponding donor fragment were mixed at a molar concentration ratio of 1:1:1 and added to fungal protoplasts. The transformants were grown for 4 day on MM at 35 °C with selection for *neo* resistance using geneticin (100 μg/mL), *bar* resistance using phosphinothricin (100 µg/mL), or *hph* resistance using hygromycin B (50 µg/mL). The presence of the transgene was confirmed by PCR.

### Analytical methods

Cultures were sampled frequently throughout fermentation to determine biomass, sugar uptake, and ethanol yield. Sugars (cellobiose and glucose), glycerol and ethanol in the supernatant were quantified by high-performance liquid chromatography (HPLC) with an e2695 instrument (Waters, Manchester, United Kingdom) equipped with a Waters 2414 refractive index detector and an Aminex HPX-87H column (Bio-Rad, Hercules, CA, USA) at 50 °C. The mobile phase was 5 mM H_2_SO_4_ with a constant flow rate of 0.5 mL/min.

### RNA extraction, sequencing, and data analysis

*Myceliophthora thermophila* strains were inoculated into fermentation medium containing 7.5% carbon source and cultured at 45 °C. Mycelia were collected at 3 days and 5 days of culture, immediately homogenized in liquid nitrogen, and then stored at − 80 °C. Total RNA was extracted as described previously [34]. For each condition, two biological duplicates were sequenced using the Illumina HiSeq™ 2000 platform (Illumina, San Diego, CA, USA). Data were analyzed as described previously [63]. Genes with reads per kilobase million (RPKM) value > 20, fold change > 2.0, and DESeq *P*_adj_ value < 0.05 were considered to differentially express between growth conditions.

### Sugar transport assay

For sugar transport assay, conidia of *M. thermophila* were inoculated into 100 mL fermentation medium containing 3% glucose or cellobiose to a final concentration of 5 × 10^5^ conidia/mL in a 250-mL Erlenmeyer flask. After cultivation at 45 °C and 150 rpm for 16 h, mycelia were transferred to the same fresh media. Samples were taken at 4 h for determination of sugar concentration and DCW.

### Growth test

Conidia of *M. thermophila* were collected from corresponding strains, filtered and adjusted to 1 × 10^6^ conidia/mL. Then 1 μL of the suspension was plated on Vogel’s minimal medium supplemented with 2% glucose or cellobiose at 45 °C or 55 °C for 4 days. H_2_O_2_ was added to the media to a final concentration of 1 mM when necessary.

For comparison of growth of WT and JY518 under different temperatures, conidia were inoculated into fermentation medium containing 7.5% cellobiose and cultivated at 45 °C, 150 rpm for 16 h, then placed at 55 °C. Samples were taken at different intervals for determination of DCW.

To determine the growth of WT under normal and oxygen-limited condition, conidia were inoculated into fermentation medium containing 7.5% glucose to a final concentration of 2 × 10^5^ conidia/mL in a 250-mL Erlenmeyer flask with (limited) or without (normal) a plug stopper. Samples were taken at 3 day and 5 day to determine DCW and DO level. DO was measured by a dissolved oxygen meter (Leici JPSJ-605, China).

### Determining copy numbers by RT-qPCR

To determine the copy numbers of the integrated *neo* and *bar* marker genes, fungal genomic DNAs were extracted and used as templates for RT-qPCR. The primers used are listed in Additional file 1: Table S1. The *actin* gene (MYCTH_2314852) was used as an internal control. The qPCR was performed as described previously [21, 23].

### Enzymatic activity assay

At 5-day fermentation using 7.5% glucose, mycelia of WT and JY144 strains were harvested and homogenated on ice immediately. After centrifugation at 16,000*g* and 4 °C for 20 min, the supernatants were used for ADH activity assay. Protein concentration of supernatants was determined using a Bio-Rad DC protein assay kit (Bio-Rad). The final reaction mixture was 600 μL consisting of 50 mM Tris–HCl pH 8.0, 2.5 mM NADH, 300 mM acetaldehyde and 30 μg total protein. Assays were performed at 25 °C and the consumption of NADH was immediately measured at a wavelength of 340 nm using a UV detector. Total ADH activity was defined as the amount of total protein required to consume 1 μmol NADH per minute under the above conditions.

## Supplementary information

**Additional file 1.** Additional tables.

**Additional file 2: Fig. S1.** Construction and characterization of Δ*aep* mutants. a Diagnostic PCR of the transformants using primers KO103702YZ-F/KO103702YZ-R. Δ*aep* mutants displayed a 1052 bp product while WT displayed a 786 bp product. b Four Δ*aep* mutants were randomly selected for growth assay. Plates were cultivated at 37 °C for 4 day. Ethanol production (c) and biomass (d) at 7 day fermentation were determined.

**Additional file 3: Fig. S2.** Determination of ethanol production and copy number of transformants. a Ethanol production by transformants overexpressing *ScAdh1*. b Ethanol production by transformants overexpressing *glt*-*1*. The transformants were fermented for 5 day. c Assay of *ScAdh1* copy number in transformants by RT-qPCR. d Assay of *glt*-*1* copy number in transformants by RT-qPCR.

**Additional file 4: Fig. S3.** Response of JY518 strain to (a) H_2_O_2_-induced oxidative stress and heat stress on medium using cellobiose as carbon source and (b) heat stress during fermentation on cellobiose.

**Additional file 5: Fig. S4.** Glycerol production of corresponding strain during fermentation on glucose and cellobiose at 5 day and 7 day.

**Additional file 6: Fig. S5.** Expression of genes involved in amino acid biosynthesis in WT and JY518 strain. Numbers represent RPKM ratio of JY518 strain to WT strain. Upregulated genes (ratio > 2, P value < 0.05) are shown in red boxes. Detailed data are shown in Additional file 1: Table S8.

**Additional file 7: Fig. S6**. Effect of dissolved oxygen (DO) on growth of *M. thermophila*. a Biomass of WT strain grown on non-oxygen limited (normal) and oxygen limited conditions. b DO levels of cultures in Erlenmeyer flask without (normal) or with (limited) a plug stopper. Fermentation media with 7.5% glucose were used.

## Data Availability

All data generated or analyzed during this study are included in this published article and its additional files.

## Abbreviations

ORF: Open reading frame
DCW: Dry cell weight
DO: Dissolved oxygen
CBP: Consolidated bioprocessing
CCR: Carbon catabolite repression
GO: Gene Ontology
WT: Wild type
