# Supplementary figures and images for: Metabolic engineering of the cellulolytic thermophilic fungus Myceliophthora thermophila to produce ethanol from cellobiose
Source: Biotechnol Biofuels. 2020 Feb 1;13:23. doi: 10.1186/s13068-020-1661-y (PMC6995234; doi:10.1186/s13068-020-1661-y)

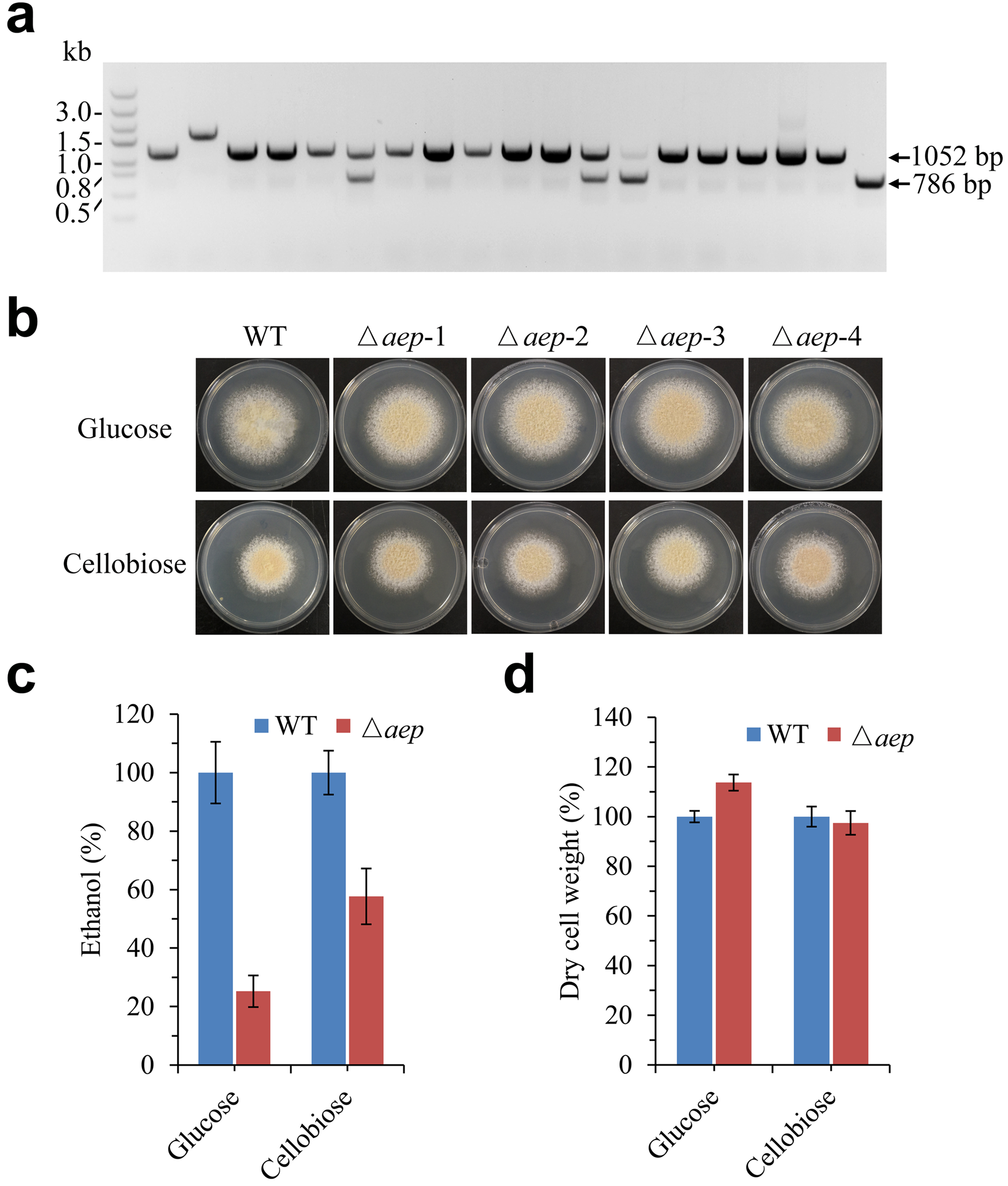

Supplement: Supplementary file 2 — Additional file 2: Fig. S1. Construction and characterization of Δaep mutants. a Diagnostic PCR of the transformants using primers KO103702YZ-F/KO103702YZ-R. Δaep mutants displayed a 1052 bp product while WT displayed a 786 bp product. b Four Δaep mutants were randomly selected for growth assay. Plates were cultivated at 37 °C for 4 day. Ethanol production (c) and biomass (d) at 7 day fermentation were determined. [file 13068_2020_1661_MOESM2_ESM.tif]

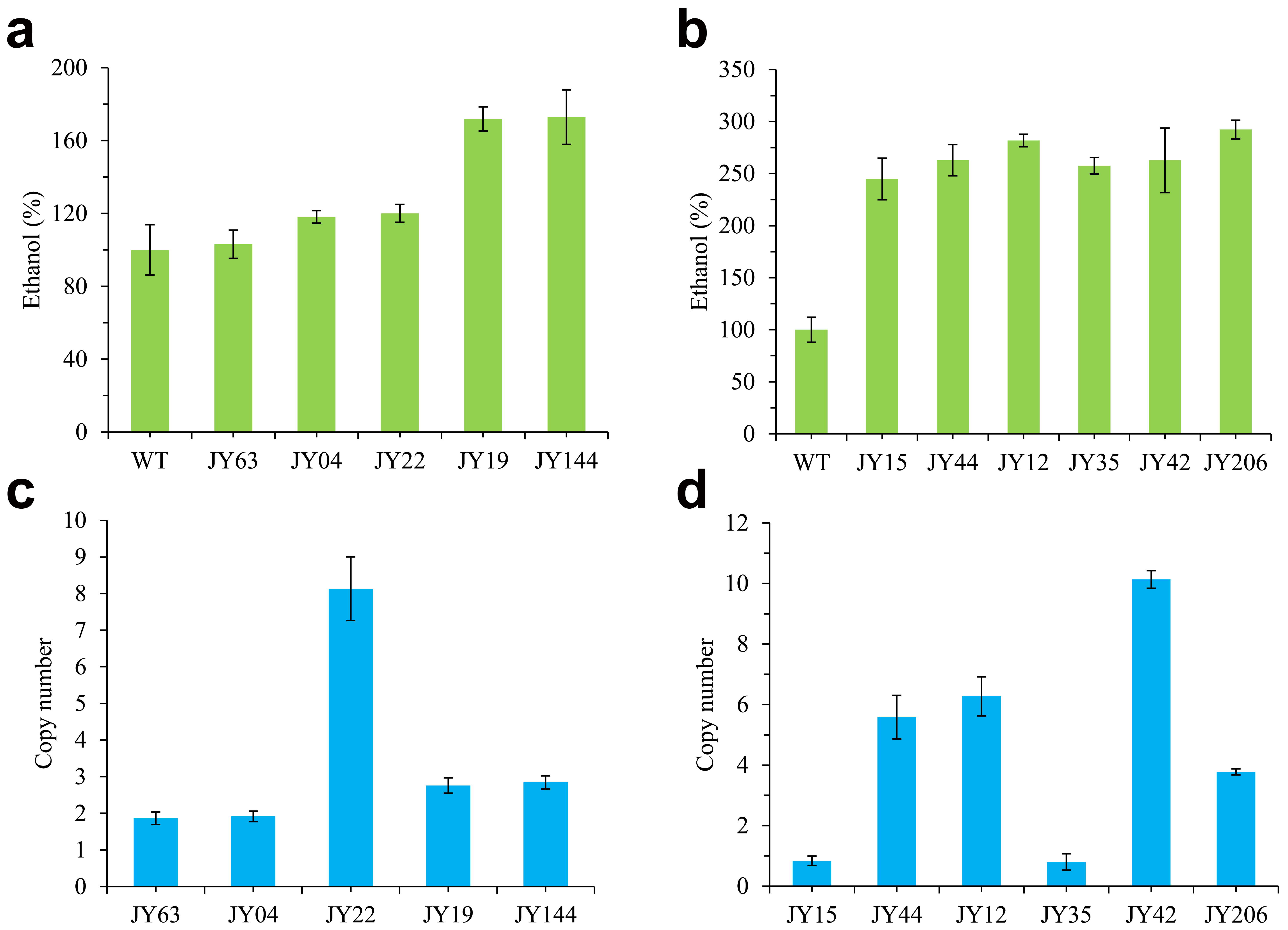

Supplement: Supplementary file 3 — Additional file 3: Fig. S2. Determination of ethanol production and copy number of transformants. a Ethanol production by transformants overexpressing ScAdh1. b Ethanol production by transformants overexpressing glt-1. The transformants were fermented for 5 day. c Assay of ScAdh1 copy number in transformants by RT-qPCR. d Assay of glt-1 copy number in transformants by RT-qPCR. [file 13068_2020_1661_MOESM3_ESM.tif]

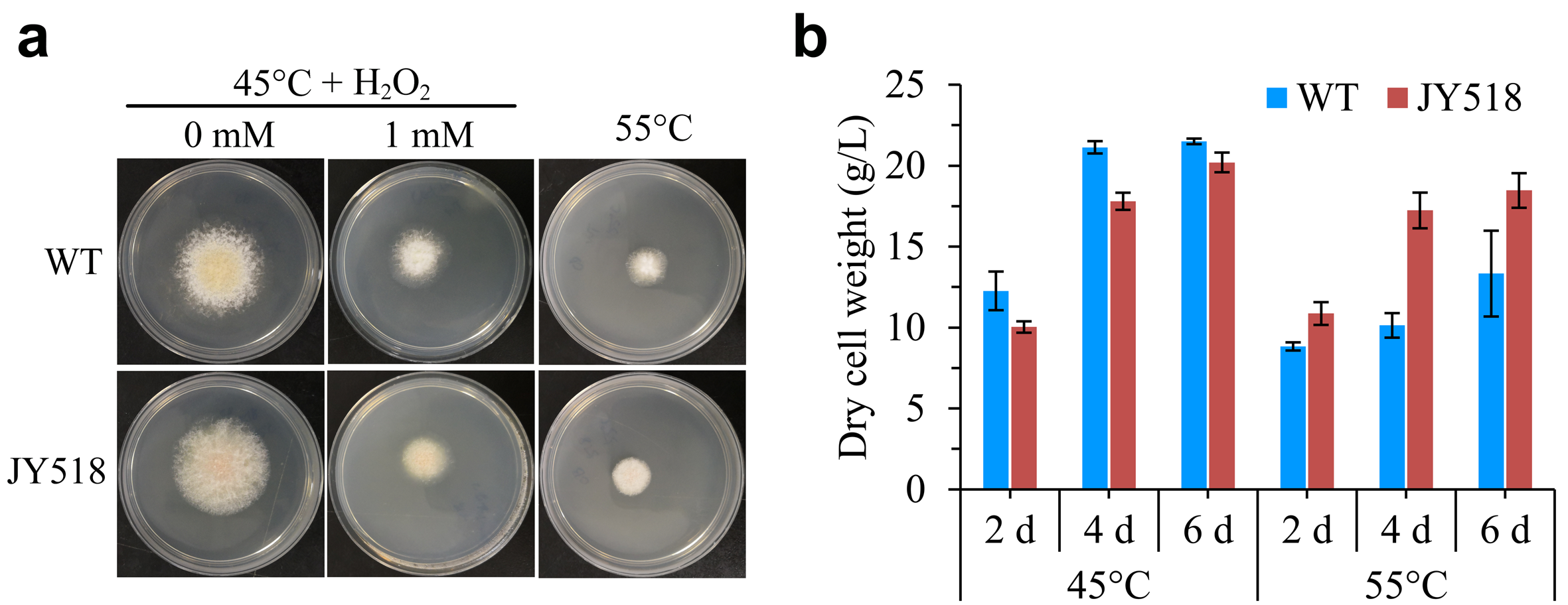

Supplement: Supplementary file 4 — Additional file 4: Fig. S3. Response of JY518 strain to (a) H2O2-induced oxidative stress and heat stress on medium using cellobiose as carbon source and (b) heat stress during fermentation on cellobiose. [file 13068_2020_1661_MOESM4_ESM.tif]

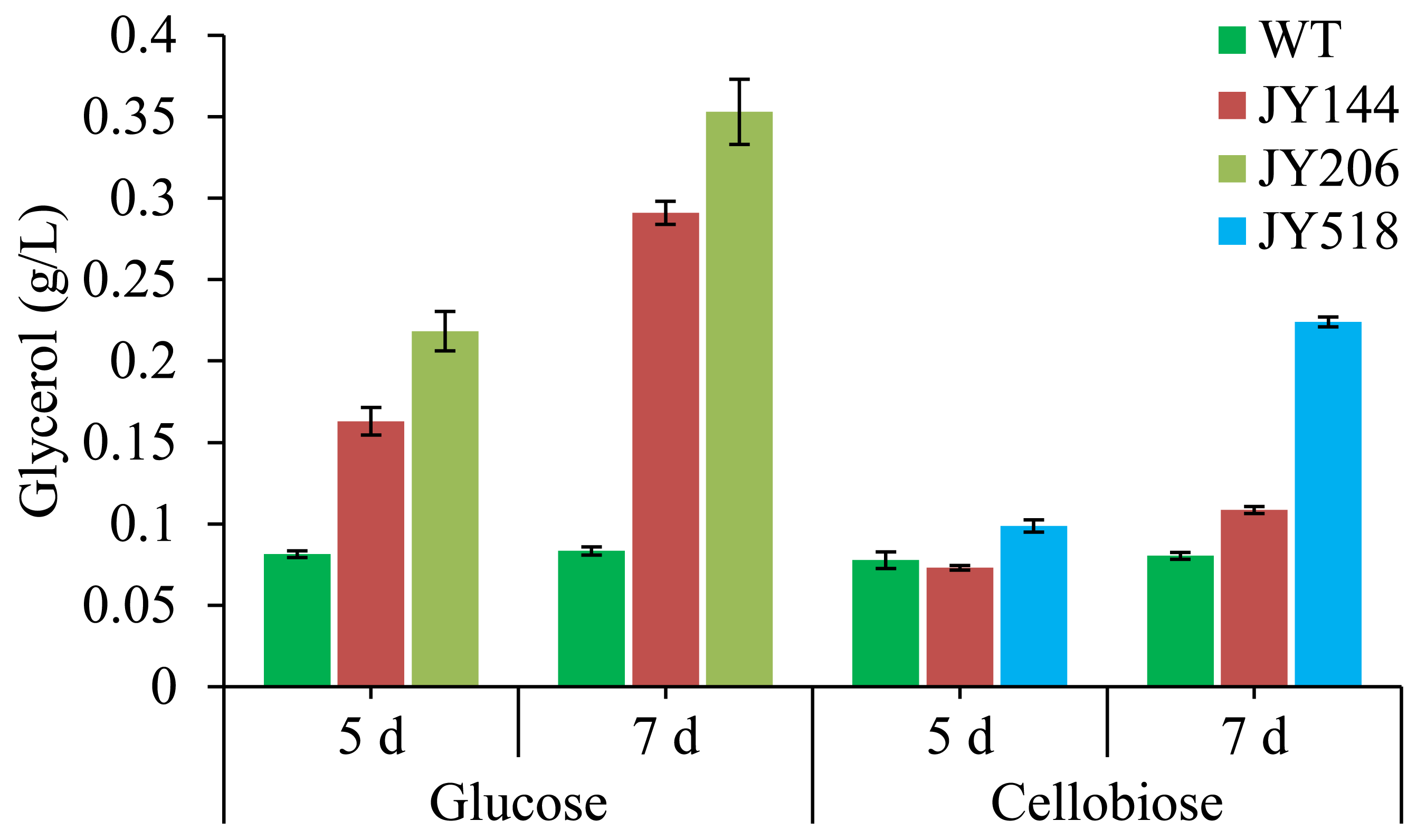

Supplement: Supplementary file 5 — Additional file 5: Fig. S4. Glycerol production of corresponding strain during fermentation on glucose and cellobiose at 5 day and 7 day. [file 13068_2020_1661_MOESM5_ESM.tif]

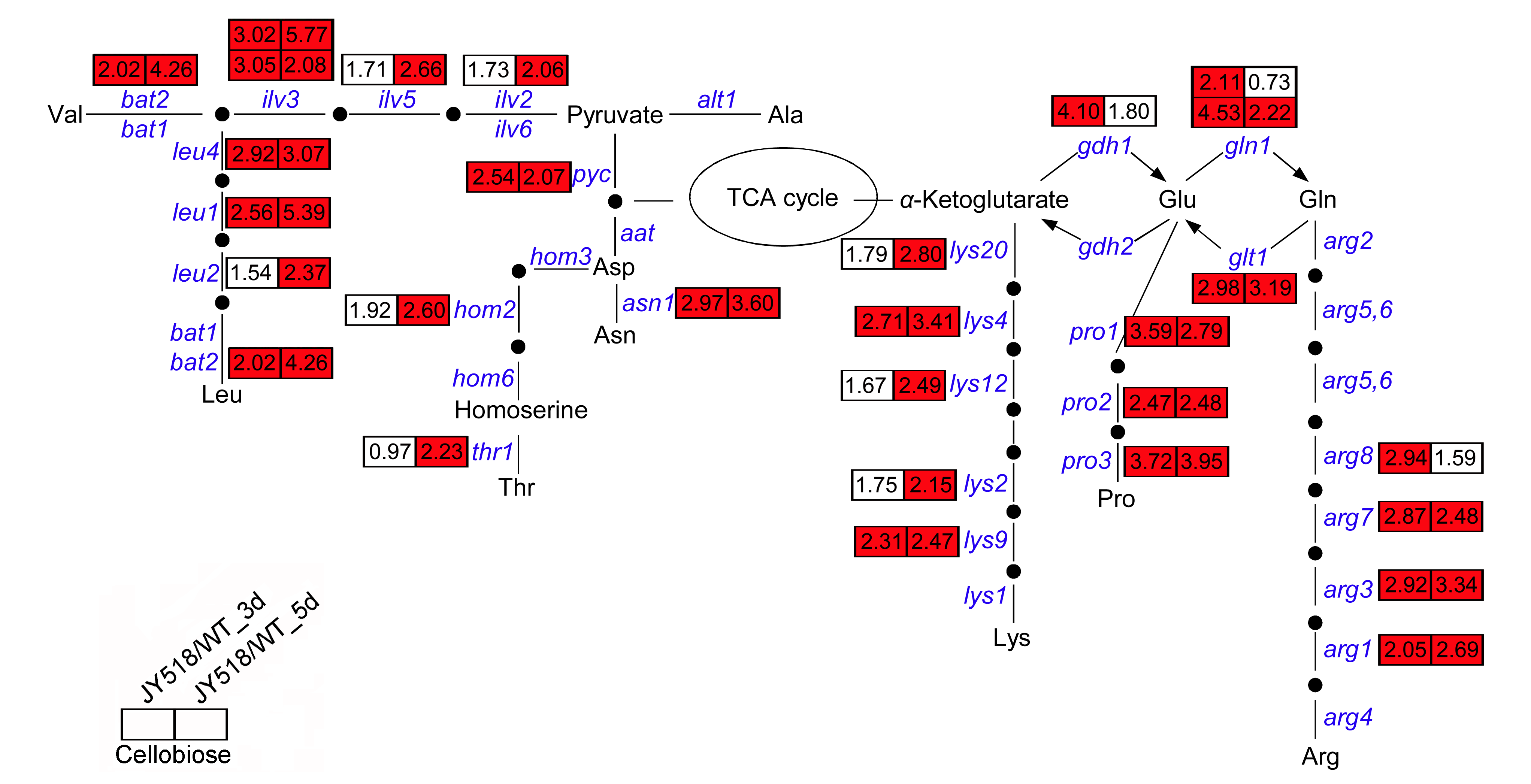

Supplement: Supplementary file 6 — Additional file 6: Fig. S5. Expression of genes involved in amino acid biosynthesis in WT and JY518 strain. Numbers represent RPKM ratio of JY518 strain to WT strain. Upregulated genes (ratio > 2, P value < 0.05) are shown in red boxes. Detailed data are shown in Additional file 1: Table S8. [file 13068_2020_1661_MOESM6_ESM.tif]

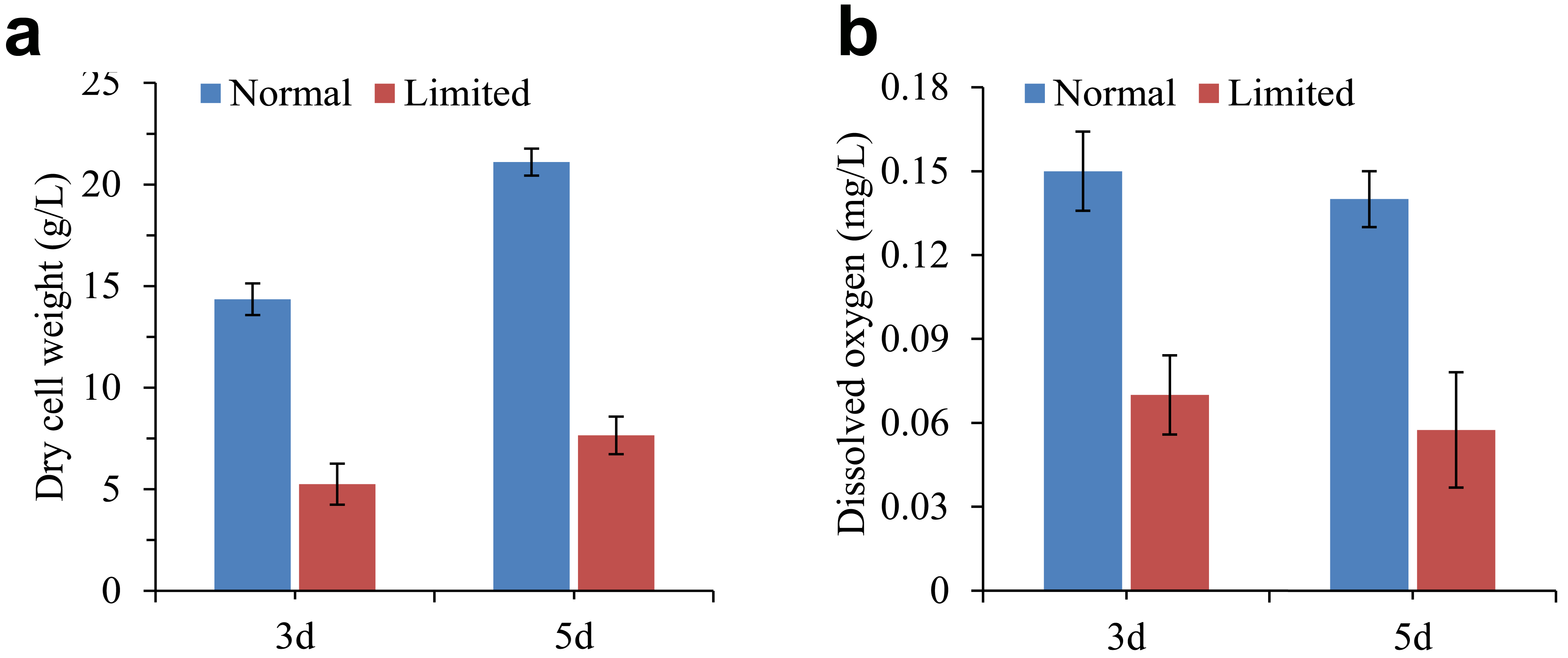

Supplement: Supplementary file 7 — Additional file 7: Fig. S6. Effect of dissolved oxygen (DO) on growth of M. thermophila. a Biomass of WT strain grown on non-oxygen limited (normal) and oxygen limited conditions. b DO levels of cultures in Erlenmeyer flask without (normal) or with (limited) a plug stopper. Fermentation media with 7.5% glucose were used. [file 13068_2020_1661_MOESM7_ESM.tif]
